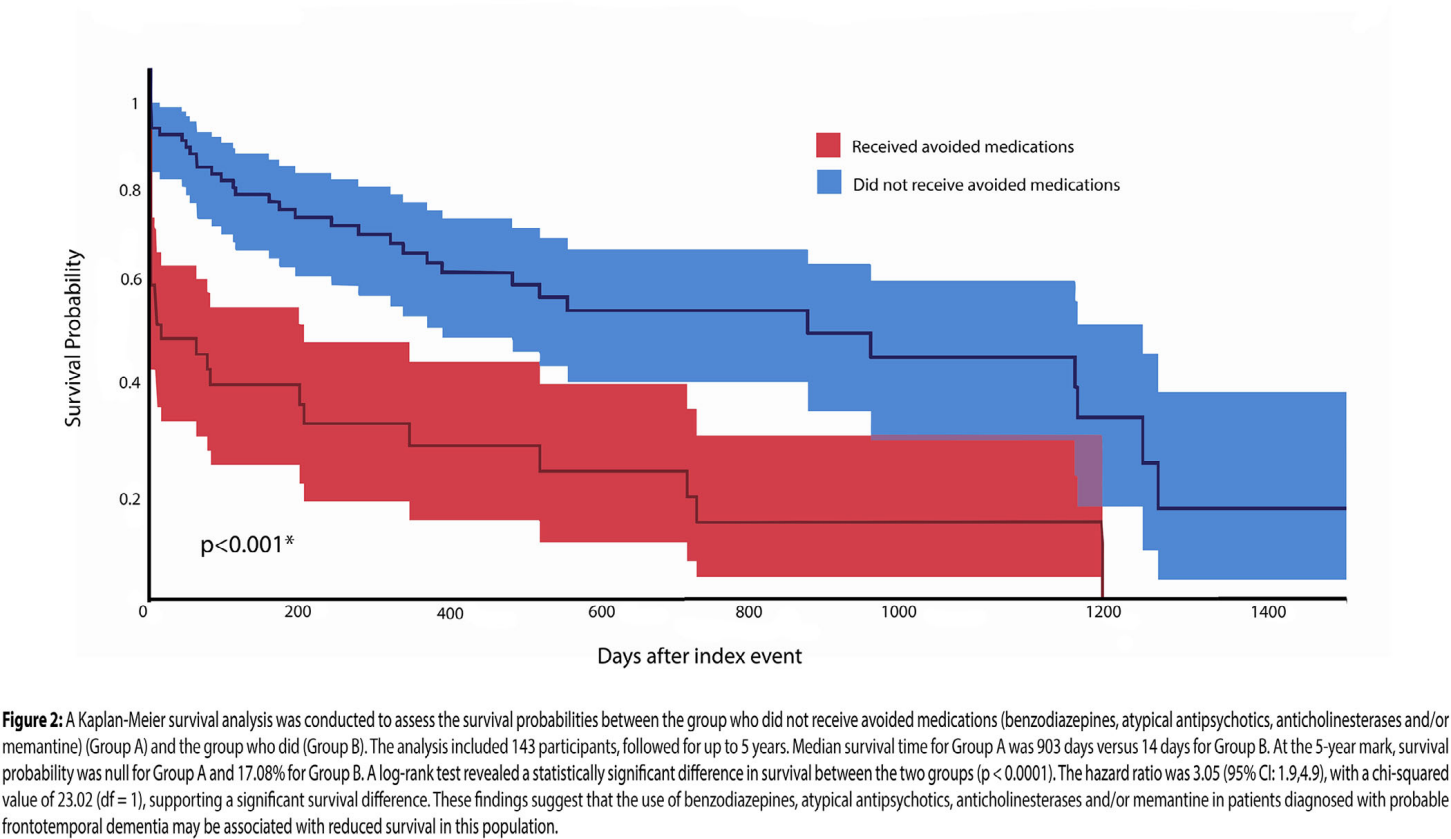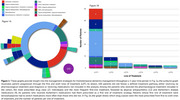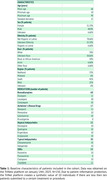# Impact of Pharmacological Management on Hospitalization Risk and Survival in Frontotemporal Dementia: A TriNetX‐based study

**DOI:** 10.1002/alz70858_107388

**Published:** 2025-12-26

**Authors:** Lorenza P. Botton, Maria Rosa Alves da Silva, Andressa de Oliveira Felício, Barbara Suman Bahlis, Eduarda Faresin, Fabricio Nery Garrafiel, Fernando Jacob Lazzaretti, Julia Patatt, Cristiano Aguzzoli, Lucas Porcello Schilling

**Affiliations:** ^1^ School of Medicine, Pontifícia Universidade Católica do Rio Grande do Sul (PUCRS), Porto Alegre, Rio Grande do Sul, Brazil; ^2^ Pontifícia Universidade Católica do Rio Grande do Sul (PUCRS), Porto Alegre, Rio Grande do Sul, Brazil; ^3^ Brain Institute of Rio Grande do Sul, PUCRS, Porto Alegre, RS, Brazil; ^4^ Instituto do Cérebro do Rio Grande do Sul, Porto Alegre, Rio Grande do Sul, Brazil; ^5^ Brain Institute of Rio Grande do Sul (InsCer), Porto Alegre, Rio Grande do Sul, Brazil; ^6^ Neurology Department, São Lucas Hospital of PUCRS, Porto Alegre, Rio Grande do Sul, Brazil

## Abstract

**Background:**

Frontotemporal dementia (FTD) is characterized by a progressive decline in behavior, executive functions, and/or language. Besides the inexistence of disease‐modifying treatments for FTD, benzodiazepines, typical antipsychotics, and Alzheimer's drugs are avoided due to ineffectiveness or harm. This study aims to identify the most commonly prescribed medications following an FTD diagnosis and compare clinical outcomes.

**Method:**

We conducted a retrospective study using data from TriNetX, a global health research network dataset from real‐world clinical settings. We included 150 individuals diagnosed with probable FTD within the last five years from 42 health centers, excluding those with other neurodegenerative conditions. All individuals underwent neuroimaging. We conducted logistic regression models and Kaplan‐Meier analysis to investigate the association and survival between medications and clinical outcomes.

**Result:**

Results showed that 144 (96%) patients came from U.S. healthcare centers. 100 individuals (67%) either received no pharmacological treatment post‐diagnosis or received medications not included in the analysis (Table 1). Benzodiazepines were the most prescribed drug class (25 individuals) and the most frequent first‐line treatment, followed by atypical antipsychotics (12) and Alzheimer's medications (9). All patients who received Alzheimer's medications had them prescribed as a first line of treatment strategy. Logistic models indicated an elevated risk of either hospitalization, emergency department visit, or critical care needs for individuals treated with benzodiazepines, typical antipsychotics, or Alzheimer's drugs (OR 4.1; 95% CI: 1.8‐8.9, *p* = 0.0002) compared to those individuals not on these treatments. Survival analysis further revealed a significant difference in survival between groups (HR 3.05 95% CI: 1.886‐4.931, *p* <0.0001; Figure 2).

**Conclusion:**

Individuals treated with benzodiazepines, typical antipsychotics, or Alzheimer's medications present a higher risk of hospitalization or critical care needs and poorer survival outcomes. Altogether, these findings suggest that these medications may be associated with adverse healthcare outcomes and reduced survival in this population. Better‐informed prescribing practices could lead to more effective patient care, brain health equity, and improved outcomes in FTD treatment.